# *Pichia pastoris* secreted peptides crossing the blood-brain barrier and DSIP fusion peptide efficacy in PCPA-induced insomnia mouse models

**DOI:** 10.3389/fphar.2024.1439536

**Published:** 2024-10-08

**Authors:** Xiaoxiao Mu, Lijun Qu, Liquan Yin, Libo Wang, Xiaoyang Liu, Dingxi Liu

**Affiliations:** ^1^ Department of Neurology, China-Japan Union Hospital of Jilin University, Changchun, China; ^2^ Department of Rehabilitation, China-Japan Union Hospital of Jilin University, Changchun, China; ^3^ Department of Clinical medicine, Zhuhai Campus of Zunyi Medical University, Zhuhai, China

**Keywords:** Pichia pastoris, peptide-crossing-blood-brain-barrier, delta-sleepinducing peptide, neurotransmitter balance, mouse model, PCPA-induced insomnia

## Abstract

**Background:**

*Pichia pastoris*-secreted delta sleep inducing peptide and crossing the blood-brain barrier peptides (DSIP-CBBBP) fusion peptides holds significant promise for its potential sleep-enhancing and neurotransmitter balancing effects. This study investigates these properties using a p-chlorophenylalanine (PCPA) -induced insomnia model in mice, an approach akin to traditional methods evaluating sleep-promoting activities in fusion peptides.

**Aim of the study:**

The research aims to elucidate the sleep-promoting mechanism of DSIP-CBBBP, exploring its impact on neurotransmitter levels and sleep regulation, and to analyze its composition and structure.

**Materials and methods:**

Using a PCPA-induced insomnia mouse model, the study evaluates the sleep-promoting effects of DSIP-CBBBP. The peptide’s influence on neurotransmitters such as 5-HT, glutamate, dopamine, and melatonin is assessed. The functions of DSIP-CBBBP are characterized using biochemical and animal insomnia-induced behavior tests and compared without CBBBP.

**Results:**

DSIP-CBBBP demonstrates a capacity to modulate neurotransmitter levels, indicated by changes in 5-HT, glutamate, DA, and melatonin. DSIP-CBBBP shows a better restorative effect than DSIP on neurotransmitter imbalance and the potential to enhance sleep.

**Conclusion:**

The study underscores DSIP-CBBBP potential in correcting neurotransmitter dysregulation and promoting sleep, hinting at its utility in sleep-related therapies.

## Introduction

In the realm of human health, sleep is pivotal, serving not only as a period for energy storage and nervous system recuperation but also as a critical phase for reinforcing brain connections, thereby augmenting learning and memory processes (Girardeau and Lopes-Dos-Santos, 2021; Kapsi et al., 2020). Despite its significance, the pervasive issue of insomnia plagues a substantial portion of the population, ushering in a host of severe health complications including depression (Riemann et al., 2020), heart disease (Frøjd et al., 2021), high blood pressure (Maiolino et al., 2021), and obesity (Knowlden and Grandner, 2021). The most prevalent remedial approach involves the administration of sedative and hypnotic drugs like benzodiazepines, yet these are not devoid of adverse effects such as impaired cognitive function (Nader and Gowing, 2020), memory loss (Al-Kuraishy et al., 2023) and perilous potential for tolerance and addiction (Rourke and Law, 2021).

Addressing this challenge necessitates a paradigm shift in sleep regulation strategies, focusing on the development of therapeutic agents that prioritize safety and efficacy. Delta sleep-inducing peptide (DSIP) is composed of 9 amino acids and has been implicated in the regulation of sleep, modulation of stress (Morgan, 2023), and exhibits a wide range of physiological and pharmacological effects. Additionally, DSIP’s functionality relies on its ability to cross the blood-brain barrier (BBB). Therefore, the use of a crossing BBB peptide (CBBBP), such as GGGGYGRKKRRQRRR, has garnered considerable attention in the scientific community for its ability to facilitate the delivery of therapeutic targets (Becker et al., 2004). This capability is particularly valuable for peptides that would otherwise struggle to effectively penetrate the BBB, thereby enhancing their therapeutic efficacy. This is a significant trait for therapeutic peptides that need to act within cells or across the BBB. The fusion with peptide crossing BBB is likely an attempt to create a more robust and easily functional form of DSIP. The fusion peptide likely retains the biological activity of DSIP. The novel agent CBBBP-and-DSIP (DSIP-CBBBP), derived through the secretion of the yeast *Pichia pastoris*, emerges as a promising candidate in this regard, with its sedative and hypnotic effects garnering significant interest.

In the specific context of sleep promotion and nervous system protection, the spotlight turns to endogenous peptides. The adoption of the PCPA-induced insomnia model in mice has enabled researchers to delve into the molecular mechanisms underlying sleep regulation (Hong et al., 2020), exploring the intricate interplay of cytokines (Wang et al., 2022), neurotransmitters (Lv et al., 2021), and their receptors. This exploration is further enriched by the sophisticated techniques of high-performance liquid chromatography and electrospray, which facilitate the meticulous analysis of polypeptide sequences and amino acid sequences, thereby unraveling the profound relationship between peptide structure and physiological function in the realms of sedation and hypnosis.

GABA (Gamma-Aminobutyric Acid) primarily functions as an inhibitory neurotransmitter in the brain, promoting relaxation and sleep (Hepsomali et al., 2020). Oral intake of GABA supplements may improve sleep quality by promoting relaxation and reducing anxiety, potentially beneficial for individuals with insomnia (Yoto et al., 2012; Abdou et al., 2006). GABA supplementation has shown potential in reducing sleep latency and improving sleep duration, suggesting it might aid in falling asleep faster and staying asleep longer (Yamatsu et al., 2015). Overall, oral GABA intake appears to offer sleep benefits by enhancing relaxation and potentially alleviating sleep disturbances (Abdou et al., 2006).

## Materials and methods

Secretory expression of the fusion peptides between Delta-sleep inducing peptides and cross the blood-brain barrier peptides (DSIP-CBBBP) from *P. pastoris*. The design of the sleeping fusion peptide plasmid is a strategic approach to creating a novel biological tool that combines the capabilities of cell-penetrating peptides, a fluorescent marker, and a DSIP for targeted research applications, particularly in the study of sleep mechanisms and potentially therapeutic interventions. The design integrates several key components, as outlined below: the chosen CBBBP is Tat, identified through prior animal experimentation from two candidate peptides for its superior efficacy in facilitating the transport of therapeutic agents crossing BBB. The amino acid sequence of Tat is YGRKKRRQRRR (Chen et al., 2022; Tyagi et al., 2006). CPPs like Tat are known for their ability to facilitate the intracellular delivery of various molecules, including peptides and proteins, which is crucial for ensuring the bioavailability of the linked therapeutic or marker proteins within cells. Linker Peptides: To connect the functional domains of the construct (Tat with DSIP), a short, flexible linker sequence GGGGS is introduced. This choice is based on the need for a linker that provides sufficient flexibility and space between the domains to ensure proper folding and functionality. The sequence GGGGS is often used in molecular biology for these purposes due to its ability to act as a flexible hinge, reducing steric hindrance and allowing the individual domains to function optimally (Hashemi et al., 2022). Fusion Peptide Sequence Design: between CBBBP and DSIP, additional GGGGS linkers are inserted to maintain flexibility and functional separation of the domains. Plasmid Vector Selection: the choice of pPICZalpha A vector from Invitrogen for the construction of this plasmid is strategic for several reasons: It is designed for high-level expression in *P. pastoris*, a yeast system that offers advantages for the production of recombinant proteins, including proper folding and post-translational modifications. The alpha factor secretion signal present in the vector facilitates the secretion of the expressed fusion peptide into the medium, simplifying purification and potential therapeutic applications. The vector also includes a Zeocin resistance gene for selection, and the presence of an AOX1 promoter allows for the methanol-inducible expression of the inserted gene, offering control over the expression levels.

Plasmid construction: pPICZαA-DSIP-CBBBP plasmid was constructed by Suzhou Hongxun Clean Technology Co. Ltd, inserting the DSIP-CBBBP gene sequence between the XhoI and XbaI sites. Meanwhile, pPICZαA-CBBBP, and pPICZαA-DSIP plasmids were also constructed by using the same way. Expansion and Transformation: These plasmids were expanded in *E. coli*, linearize via *Sal*I, and subsequently transformed into *P. pastoris* cells via electroporation. The transformed Pichia pastoris cells are induced to express these proteins by adding 0.5% methanol daily to the culture, respectively. The secretory expressed proteins were subjected to SDS-PAGE to confirm their size and purity.

### Experimental animals

A total of 48 Kun-Ming strain male mice were used. The mice were specific pathogen-free (SPF) and about 8 weeks old, weighing 20 ± 2 g. They were housed under controlled laboratory conditions with a 12h/12 h light/dark cycle, at a constant temperature of 25°C ± 1°C and relative humidity of 60% ± 10%. Throughout the study, the mice had free access to standard laboratory granular diets and distilled water. All procedures were conducted following the ethical standards and guidelines for laboratory animals set by the People’s Republic of China and the Declaration of Helsinki. The animal protocol was specifically approved by the Scientific Ethics Committee of Ethics Committee of the School of Public Health, Jilin University (approval No. 2023-11-06).

### Insomnia model establishment

The insomnia model was established by PCPA at a dose of 300 mg/kg via i.p. injection for five consecutive days, commencing on the eighth day of the study. Insomnia was confirmed by monitoring sleep disturbances and alterations in neurotransmitter levels using high-performance liquid chromatography (HPLC). This comprehensive experimental design ensures controlled conditions, adequate sample size, and systematic data collection, allowing for the evaluation of DSIP’s efficacy in ameliorating neurotransmitter imbalance and enhancing sleep in PCPA-induced insomnia mouse models.

The experimental animal, properly identified by its weight number, should be securely restrained by an assistant, positioning the animal with its head downward and abdomen upward. During the process of intraperitoneal injection, the injector, holding the syringe in the right hand, should utilize the left hand’s little and ring fingers to grasp the mouse’s tail firmly. The remaining fingers should secure the mouse’s neck, facilitating the rotation of the mouse to position its head downwards and abdomen upwards. The syringe needle is then to be inserted subcutaneously into the lower left abdominal area at a 45-degree angle through the abdominal muscles. Care must be taken to ensure the needle does not penetrate too deeply to prevent potential organ damage. A successful needle insertion is indicated by a clear sense of penetration and a somewhat resistant retraction, with only air being aspirated back into the syringe. Once the needle is securely in place, the medication should be administered slowly. The insertion technique should be executed with gentleness to avoid harming the abdominal organs. Post-injection, the needle should not be immediately withdrawn but rather left *in situ* within the peritoneal cavity for an additional duration to effectively seal the puncture site and prevent medicinal leakage, ensuring the medication disperses effectively with the organs’ movements. The experimental animal’s condition should be meticulously observed following the procedure. The administered concentration of PCPA was set at 300 mg/kg via i.p. injection for five consecutive days, commencing on the eighth day of the study. Notable observations post-administration include a marked increase in daytime activity, the erasure of circadian rhythms (Choudhury et al., 2022), a diminution in fur sheen (Lavigna et al., 2021), widespread hair loss among the subjects, escalated aggression, and occurrences of mutual biting (Huang et al., 2024).

### Pharmacological intervention

Experimental groups were established, each comprising 6 model mice and 6 control mice. The animals were restrained with their heads positioned downward and abdomens upward for the i.p. injection procedure. Anesthesia was administered prior to drug injections to ensure animal comfort and minimize stress. Isoflurane (2%–3% concentration inhaled in oxygen) was used as the anesthetic agent, with a carrier gas flow rate set at 0.2–0.5 L per minute (L/min) for induction and maintenance. The total duration of anesthesia was approximately 10 min, and injections were conducted at room temperature (∼22°C). The drugs (GABA, DSIP, DSIP-CBBBP, and CBBBP) were administered via i.p. injection at a dosage of 100 nM, prepared in a saline solution concentration of 10 mM/L. Control mice received an equivalent volume of saline solution. Injections were administered for five consecutive days to the respective experimental groups, starting on the eighth day of the study. Post-injection, the needle remained in the peritoneal cavity for a period to ensure effective sealing of the injection site, preventing medication efflux and facilitating absorption during peristalsis. The animals were carefully observed post-intervention for any adverse effects or behavioral changes.

### Open field test (OFT)

The OFT assesses spontaneous locomotor activity and exploratory behavior in experimental animals. This test was conducted both before and after the 5-day PCPA administration period. Prior to the experiment, the experimental box should be thoroughly cleaned and free of odors, with particular attention paid to removing any feces and urine from previous tests at the bottom of the box. Experimenters should set up the corresponding parameters in the software, including the animal’s number, date, and status light information. The experimental animal should be gently removed from the breeding cage, ensuring it faces away from the experimenter, then quickly placed in the central area of the experimental box before the experimenter promptly exits. The animal behavior analysis software should be initiated to automatically record the animal’s activity within the box, typically for a duration of 15 min. Following the experiment, the animal should be moved to a previously prepared breeding cage. The apparatus should be deodorized with alcohol spray and wiped dry with paper towels. To minimize the impact of nonspecific stress stimuli on the experimental animals, they should be handled for 1–2 min daily before the experiment. The experiment requires dim lighting, and observers should maintain as much separation from the test animals as possible. The environment should remain quiet throughout the test. For the open field test, experimental animals need to be screened; animals showing significantly lower levels of activity than the average level of the cohort should be excluded beforehand.

### Elevated plus maze (EPM)

The elevated plus maze evaluates anxiety-like behavior by measuring the animal’s exploration of open and enclosed arms. This test was conducted following the 5-day PCPA administration period. Animals should be allowed to explore the apparatus for about 10 min, with white noise played to eliminate distracting sounds. At the beginning of the test, animals should be placed in a closed quadrant. They may spend time either in the closed area or in the open area. The experiment should be recorded for 5 min. Behavioral measurements should primarily include the percentage of time spent in the open quadrant, the percentage of entries into the open quadrant, the number of times the animal peeks down from the edge of the platform while in the open or closed quadrant, and the number of stretch-attend postures from the closed quadrant to the open quadrant. An animal is considered to be in the open area when all four paws are in an open quadrant and in the closed area only when all four paws have crossed from the open to the closed boundary.

### Tail suspension test

The tail suspension test measures depressive-like behavior by assessing the duration of immobility when the animal is suspended by its tail. This test was conducted following the 5-day PCPA administration period. Animals acclimated to the animal room for 3 days should be brought into the laboratory. A 12-h day/night cycle allows the animals to maintain a normal circadian rhythm, with free access to food and water. Animals should be gently removed from the cage and their tails quickly secured without causing undue stress. Specifically, medical tape should be used to adhere at a point 1 cm from the tail tip, allowing for the tail to be suspended from the apparatus’ hanging rod. The distance between the tip of the animal’s tail and the ground should be about 30 cm, placing the mouse in an inverted position. The camera should be adjusted in advance to aim horizontally at the mouse’s body to ensure a complete field of view in the recording. The total recording should last 6 min, capturing the animal’s behavior during the experiment, but focusing on the last 4 min of immobility time. After the recording period, the medical tape should be carefully removed from the animal, which should then be placed back in its original cage with proper documentation. Following the completion of all animal tests, the apparatus should be promptly cleaned of any feces and urine.

### Sucrose preference test

For clarity and adherence to the experimental timeline, the sucrose preference test was performed after these behavioral tests to assess the impact of PCPA-induced insomnia on sucrose preference in the animals. Before commencing the experiment, mice should be housed individually for a week to adapt to their environment. To prepare the sucrose solution and water, approximately 40 mL of water should be added to a 50 mL centrifuge tube, which is then sealed with a straw cap. The tube should be inverted to allow air bubbles to rise. When inverting, it's crucial to ensure that the water line remains below the conical end. Each tube should be labeled clearly with either ‘A' for the sucrose solution or ‘B' for pure water, or alternatively, the initial weight of the bottles can be recorded. The two inverted tubes should be carefully placed into the cage’s metal lid, avoiding any shaking to prevent dripping. Both bottles should be positioned on one side of a separate metal rack, with food placed on the opposite side. It's important to ensure that the rubber stopper and the locking device are aligned properly, with the steel straw extending below the metal cage lid. Food should be placed on the opposite side of the wire lid. Liquid flow should be monitored for any issues, and the area beneath the straws should be checked for signs of leakage, such as wet bedding. The position of the tubes should be alternated daily (left to right). The mice’s preference for sucrose is calculated as follows: % sucrose preference = (sucrose water consumption/(sucrose water consumption + pure water consumption)) × 100%, indicating the degree of the mice’s preference for sucrose.

### Wakefulness time measurement

To evaluate the impact of Pichia pastoris secreted recombinant peptides on insomnia, we analyzed the increase in wakefulness time among different groups of mice: Control, Model, GABA, DSIP-CBBBP, DSIP, and CBBBP. After establishing a 5-day PCPA-induced insomnia model, treatments were administered over a subsequent 7-day period. Columbus Instruments’ Opto-M3 (Instrument Type No. 12345) was employed, which uses a grid of infrared beams to detect and record the animal’s movements, providing precise measurements of wakefulness time.

### Hematoxylin and eosin (H&E) staining

H&E staining was performed to assess tissue morphology and structural changes in brain tissue samples. Twelve samples preserved in 4% paraformaldehyde undergo ethanol dehydration through graded concentrations (85% for 2 h, 95% for 2 h, followed by 100% for 1 h each) before being cleared in xylene and embedded in paraffin. Sections of 4 μm thickness are prepared using a paraffin microtome (HS3315, Huadu Technology, Zhejiang) and then dried in an oven (GZX-9030MBE, Boxun, Shanghai) at 60°C for 1 h. The sections are then rehydrated, leading up to the H&E staining where hematoxylin stains the nuclei blue and eosin stains the cytoplasm and extracellular matrix pink. After staining, sections undergo a series of alcohol dehydrations, clearing in xylene, and are then mounted with a neutral resin before being examined under a microscope (BDS400-FL, Ote, Chongqing) for histological evaluation. This comprehensive process, utilizing reagents and instruments from various suppliers including Shenggong (Shanghai) for xylene and paraffin, Dingguo (Beijing) for 4% paraformaldehyde, and both Shenggong and Dexinkang for alcohols, ensures a detailed morphological assessment of the brain tissue’s response to treatment. To accurately assess neuron density, five non-overlapping fields of view within each brain tissue section were selected to represent the overall tissue morphology. The number of neurons in each of these fields were counted manually. After counting, the average neuron density was calculated by dividing the total number of neurons by the five fields analyzed. Statistical analysis involved counting changes in fissures and average neuron density observed in individual brain sections analyzed per microscopy field.

### Neurotransmitter measurement

To evaluate the impact of *P. pastoris* secreted recombinant DSIP-CBBBP on neurotransmitter levels, we quantified the concentrations of melatonin, serotonin, dopamine, and glutamate in the mouse serum. Blood samples (100 μL each) were collected from mice via cardiac puncture and allowed to clot at room temperature for 30 min. The samples were then centrifuged at 2,000 g for 15 min at 4°C to separate the serum. The supernatant (serum) was carefully collected and stored at −80°C until analysis. High-performance liquid chromatography (HPLC) and enzyme-linked immunosorbent assay (ELISA) were employed to quantify the levels of these key neurotransmitters and neurotrophic factors. This comprehensive analysis aimed to elucidate the neurochemical changes associated with PCPA-induced insomnia and the potential therapeutic effects of our treatment.

For the quantification of neurotransmitters such as dopamine, serotonin, and glutamate, HPLC plays a pivotal role. This technique leverages an electrochemical detector (ECD) for dopamine and serotonin, and a fluorescence detector for glutamate, utilizing equipment from reputable companies like Shimadzu Corporation or Agilent Technologies. The chromatographic separation occurs on a reversed-phase C18 column, with the mobile phase composition and flow rates specifically tailored for each neurotransmitter. The conditions, such as a 75 mM sodium phosphate buffer (pH 3.0) with 1.7 mM 1-octanesulfonic acid, 25 µM EDTA, 10% methanol, and a flow rate of 1 mL/min, are optimized for precision and sensitivity, ensuring accurate neurotransmitter profiling at a column temperature of 25°C. ELISA assays complement the HPLC analysis by measuring concentrations of melatonin and neurotrophic factors, utilizing kits from Sangon Biotechnology Company (Shanghai, China) following strict protocols to ensure reliability. This involves preparing samples and standards in a specific diluent, followed by incubation, washing, and detection steps that culminate in absorbance measurement at designated wavelengths. The procedure is carefully controlled for temperature and timing, with incubations typically ranging from 1 to 2 h at room temperature to overnight settings at 4°C, depending on the target analyte.

### Statistical analysis

Statistical analysis of the collected data is crucial for interpreting the effects of Pichia pastoris secreted recombinant DSIP-CBBBP on neurotransmitter levels in brain tissue. After quantifying the concentrations of melatonin, serotonin, dopamine, glutamate, and neurotrophic factors using HPLC and ELISA, the results are analyzed using statistical software such as GraphPad Prism. This involves the comparison of treated and control groups to assess the significance of the treatment’s impact. Typically, an Analysis of Variance (ANOVA) is employed, possibly followed by *post hoc* tests to pinpoint specific differences between groups.

## Results

### Electrophoresis analysis of peptide expression

**FIGURE 1:**
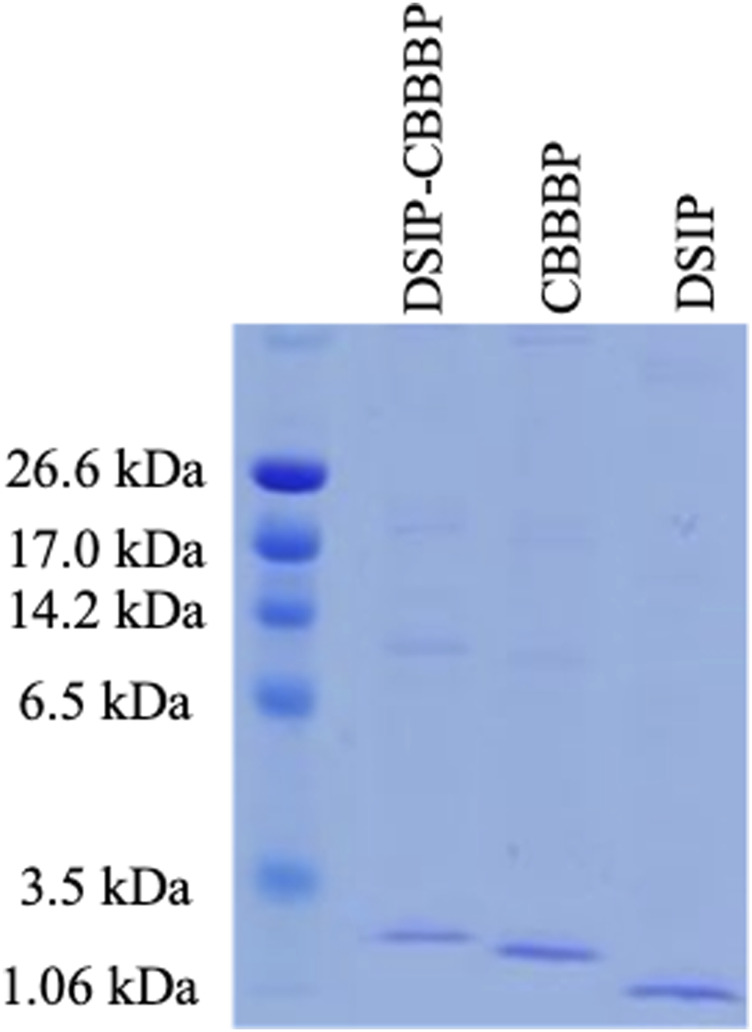
The expression of CBBBP, DSIP (Delta-Sleep Inducing Peptide) and fusion peptides DSIP-CBBBP in *Pichia pastoris*. The molecular weights of CBBBP, DSIP, and DSIP-CBBBP are 1.56 kDa, 0.88 kDa, and 2.71 kDa, respectively.

Figure 1 shows successful expression of DSIP-CBBBP, CBBBP, and DSIP. The presence of bands in the lanes corresponding to the DSIP-CBBBP, CBBBP, and DSIP constructs suggests successful expression of these fusion proteins. Assuming the bands appear at the expected positions corresponding to their theoretical molecular weights, this indicates that the fusion proteins were expressed at the correct size and were not significantly degraded. Use in Insomnia Mouse Model: The three proteins that were successfully expressed and confirmed by their molecular weight and fluorescence (DSIP-CBBBP, CBBBP, and DSIP) were used in the insomnia mouse model. The use of these proteins would be based on their expected biological activity: DSIP as the active peptide expected to induce sleep, and CBBBP to facilitate entry into brain.

### DSIP-CBBBP reduces anxiety or depressive-like states

**FIGURE 2:**
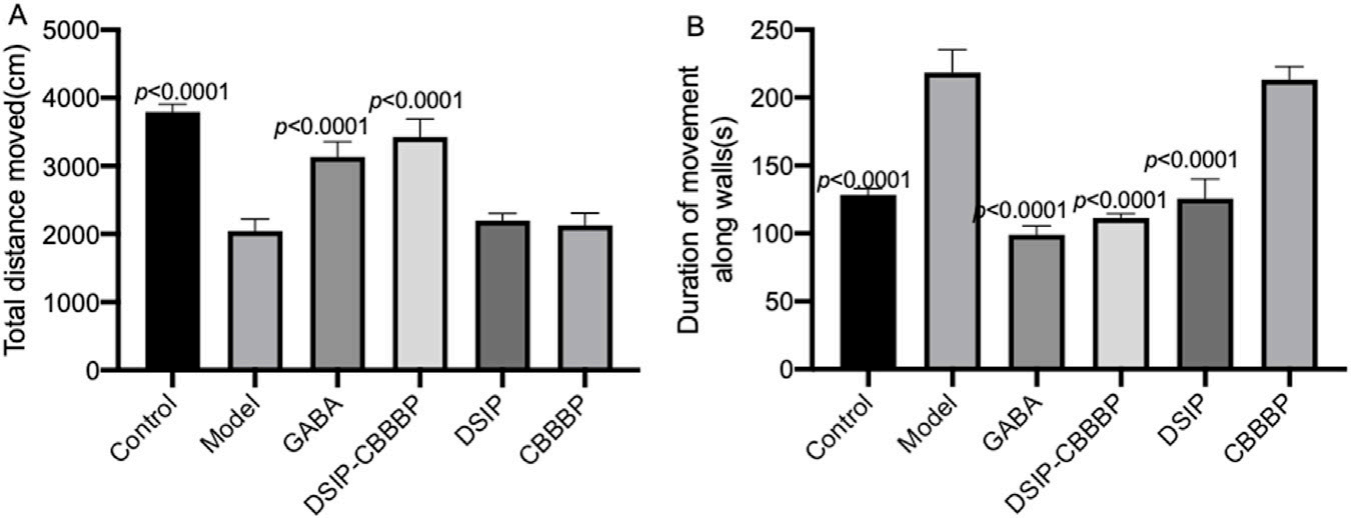
Valuation of movement and activity in insomnia mouse model following treatment with fusion proteins. **(A)** total distance moved by mice in open field test. **(B)** time spent moving in open field test by mice. N = 6 for each group. The statistical difference is significant if *p* < 0.05 via the model group.

Figure 2 delineates the effects of different treatments on locomotor activity in mice within an insomnia model, as assessed by the open field test. The total distance moved (Figure 2A, F (5, 30) = 101.8, *p* < 0.0001) and the time spent moving (Figure 2B, F (5, 30) = 148.6, *p* < 0.0001) serve as indicators of exploratory behavior and general activity, with potential implications for anxiety or depressive-like states. In Figure 2A, the control group establishes the baseline level of activity, contrasted with the model group, which displays a significant decrease in movement, indicative of the behavioral suppression typical in insomnia (*p* < 0.0001). The administration of GABA is associated with an increased total distance moved (*p* < 0.0001), suggesting its effectiveness in counteracting the reduced mobility observed in the insomnia model. The DSIP-CBBBP and DSIP treatments show a statistically significant enhancement in the distance moved (*p* < 0.0001 for DSIP-CBBBP and DSIP), suggesting their potential in ameliorating the locomotor deficits associated with insomnia. CBBBP alone, while resulting in an increase in distance, does not have its *p*-value explicitly stated, thereby not clearly defining its statistical impact. Figure 2B complements these findings by showing the total time spent moving. The control group’s activity time is significantly higher than that of the model group, which shows a marked decrease (*p* < 0.0001), consistent with increased levels of anxiety or depression. Treatment with GABA significantly increases activity time (*p* < 0.0001), highlighting its anxiolytic properties. Similarly, DSIP-CBBBP and DSIP treatments induce significant increases in movement time (*p* < 0.0001 for both), indicating their potential in reducing depressive-like behaviors. CBBBP treatment also shows an increase in activity time, but like in Figure 2A, the absence of a *p*-value prevents a definitive assessment of its statistical significance.

The OFT results emphasize that sleep quality was significantly affected in the insomnia model, as evidenced by decreased locomotor activity and movement time. The model group, which experienced induced insomnia, displayed marked reductions in both total distances moved and time spent moving, reflective of heightened anxiety and depressive-like states. Treatments with GABA, DSIP-CBBBP, and DSIP significantly mitigated these deficits, restoring locomotor activity and movement time to levels closer to those observed in the control group. Specifically, DSIP-CBBBP demonstrated robust effects in reducing anxiety and depressive-like behaviors, underscoring its therapeutic potential in improving sleep quality and overall behavioral health in the context of insomnia.

### DSIP-CBBBP reduces anxiolytic status

**FIGURE 3:**
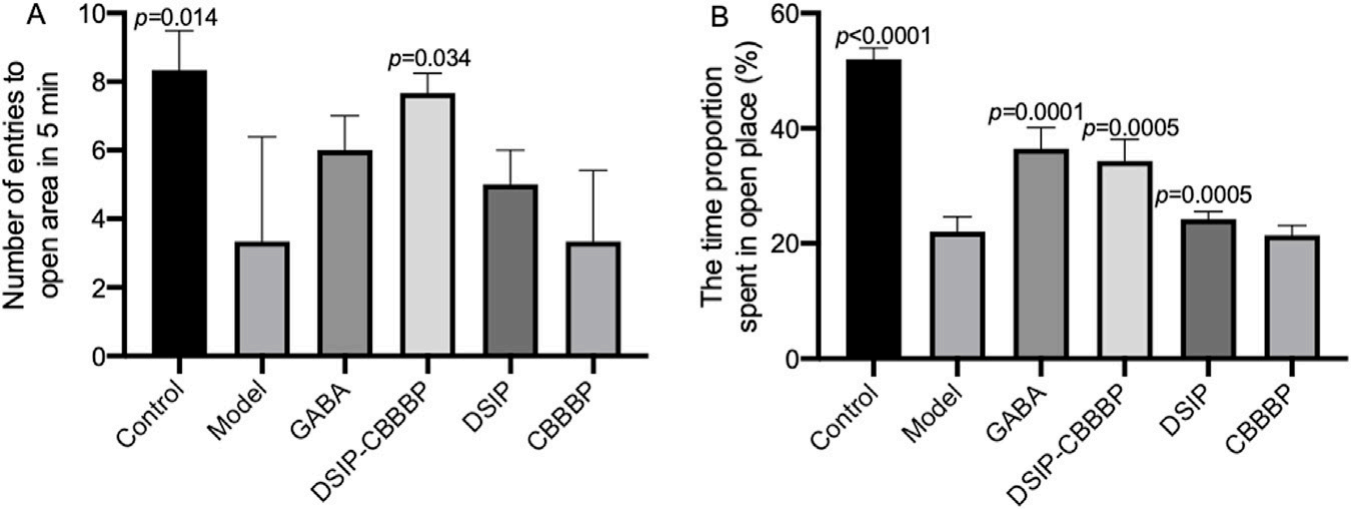
Assessment of anxiolytic effects in mice using the elevated plus maze (EPM) test. **(A)** number of entries into open arms. This graph represents the number of times mice entered the open arms of the EPM, a measure of exploratory behavior and anxiety levels. **(B)** proportion of time spent in open arms. This graph quantifies the proportion of time spent in the open arms during the EPM test, directly correlating with the level of anxiety-like behavior in mice. N = 6 for each group. The statistical difference is significant if *p* < 0.05 via the model group.

Figure 3 provides an insightful exploration of anxiolytic effects in mice through the use of the EPM test, with a sample size of N = 6 per group, examining both the frequency of entries into open arms (Figure 3A, F (5, 30) = 5.68, *p* = 0.013) and the proportion of time spent in these arms (Figure 3B, F (5, 30) = 58.36, *p* < 0.0001), which are indicative of anxiety levels in rodents. In Figure 3A, the number of entries into open arms of the EPM by the control group suggests a baseline level of exploratory behavior and innate anxiety, with the model group showing significantly fewer entries (*p* = 0.014), indicative of higher anxiety levels induced by the experimental conditions. The GABA treatment group, however, does not demonstrate a significant difference from the model group (*p* = 0.254), implying that under these experimental parameters, GABA may not exert a strong anxiolytic effect on this specific measure of anxiety. The DSIP-CBBBP group exhibits a significant increase in open arm entries compared to the model (*p* = 0.034), suggesting a reduction in anxiety levels, while the DSIP group shows no significant difference (*p* = 0.646), and CBBBP alone indicates no change (*p* = 0.999) compared to the model group. These results hint at a nuanced effect of the DSIP-CBBBP treatment on anxiety, possibly attributed to its combined peptide and DSIP components, which may modulate anxiety differently than when DSIP is used in isolation with CBBBP.

Figure 3B further elucidates these behaviors by measuring the time proportion spent in the open arms, with the control group spending the most time in the open arms, aligning with lower anxiety-like behavior. The model group’s significantly less time spent in the open arms (*p* < 0.0001) underscores the success of the anxiety induction in this model. The GABA treatment group shows a significant increase in the time spent in the open arms (*p* < 0.0001), reinforcing its anxiolytic properties. Both DSIP-CBBBP and DSIP treatments result in a significant increase in time spent in the open arms (*p* < 0.0001 and *p* = 0.0005, respectively), suggesting their potential anxiolytic effects. However, CBBBP alone does not produce a significant change (*p* = 0.999), indicating that the DSIP component may be critical to the anxiolytic activity observed in the DSIP-CBBBP and DSIP groups. These findings suggest that the molecular configuration of the treatments influences the behavioral outcomes and that the presence of DSIP, particularly in combination with a CBBBP, may be crucial for the observed anxiolytic effects.

These findings highlight that by reducing anxiety levels, DSIP-CBBBP treatment significantly enhances sleep quality. The significant reduction in anxiety, as evidenced by the increased entries and time spent in the open arms of the EPM, suggests that DSIP-CBBBP effectively alleviates the stress and anxiety associated with insomnia. This reduction in anxiety likely leads to improved sleep quality, addressing both the psychological and physiological components of sleep disturbances in the insomnia model. Thus, the DSIP-CBBBP treatment shows great promise in mitigating the adverse effects of insomnia on sleep quality.

### DSIP-CBBBP and DSIP reduce depressive-like behaviors

**FIGURE 4:**
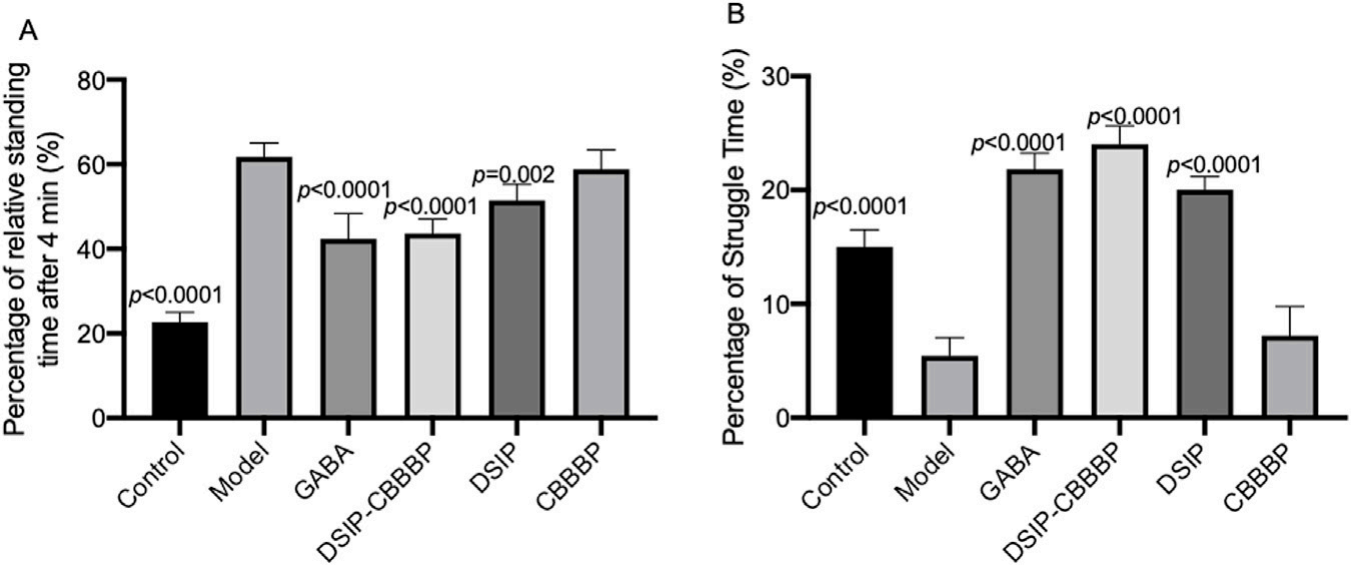
Effects of different treatments on depressive-like behaviors in the tail suspension test. **(A)** immobility time. The graph shows the percentage of time that animals spent immobile during the tail suspension test. **(B)** Struggle time. This graph depicts the percentage of time animals engaged in struggle behaviors during the tail suspension test. N = 6 for each group. The statistical difference is significant if *p* < 0.05 via the model group.

Figure 4 presents the outcomes of a tail suspension test used to assess depressive-like behaviors in an animal study. This test measures the duration of immobility and struggle time as indicators of depressive behaviors, with the premise that longer periods of immobility and shorter struggle times correlate with more severe depressive-like states. In Figure 4A (F (5, 30) = 106.4, *p* < 0.0001), the control group exhibits the expected baseline behavior with the lowest immobility percentage. The model indicates a significant increase in immobility time (*p* < 0.0001), suggesting the induction of an insomnia induced depressive-like state. The model treated with GABA, also shows a significant reduce in immobility when compared to the model group (*p* < 0.0001). DSIP-CBBBP presents a reduced immobility time when compared with the model group (*p* < 0.0001), implying the reduction of depressive-like behavior. DSIP but not CBBBP follow suit, demonstrates significant reduction in immobility (*p* = 0.002) and is indicative of reduced depressive-like behavior compared to the model but less than DSIP-CBBBP effects. Figure 4B captures the percentage of struggle time (F (5, 30) = 59.88, *p* < 0.0001), where the control has the highest struggle percentage, aligning with the least depressive-like behavior. The model shows a substantial reduction in struggle time (*p* < 0.0001), which is consistent with a depressive-like condition. GABA has a longer struggle time when compared with the model (*p* < 0.0001) as well, indicating a possible depressive effect. DSIP-CBBBP and DSIP exhibits an increase in struggle time when compared with model (*p* < 0.0001), suggesting that both treatments may influence depressive-like behaviors but not CBBBP alone.

### DSIP-CBBBP and DSIP increase sucrose preference

**FIGURE 5:**
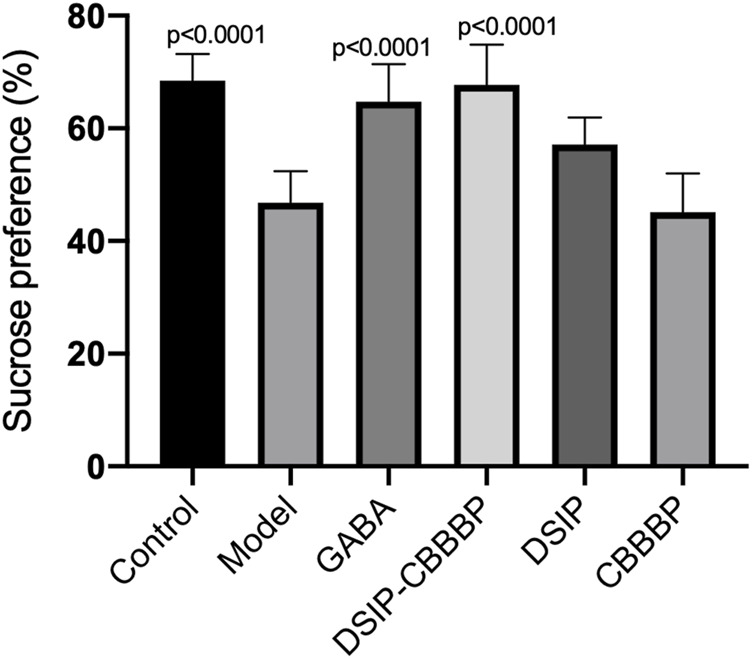
Sucrose preference among different groups. This graph represents an indirect measure of sucrose preference, which is associated with hedonic or pleasure-seeking behavior in mice. Sucrose preference is often used as an indicator of anhedonia, a symptom characteristic of emotional disorders including depression. N = 6 for each group. The statistical difference is significant if *p* < 0.05 via the model group.

Figure 5 presents the effects of various peptide treatments on sucrose preference affected by PCPA in mice. The results show a significant overall effect across the different groups (F(5, 30) = 17.83 *p* < 0.0001). The control group shows expected baseline values, while the model group displays a marked reduction in sucrose preference (*p* < 0.0001). The treatments with DSIP and DSIP-CBBBP indicate a significant improvement compared to the model group, highlighting the potential therapeutic effects of these peptides on hedonistic behavior. Further analysis reveals that the DSIP-CBBBP group shows a notable improvement compared to both the model group and the DSIP-treated group, indicating that the fusion peptide may have enhanced efficacy and exerting its therapeutic effects (*p* < 0.0001). The CBBBP alone does not show as significant an effect, suggesting that while it aids in delivery, it is the DSIP component that is primarily responsible for the observed improvements in hedonistic behavior. Overall, the results demonstrate the effectiveness of DSIP and its fusion with CBBBP in mitigating PCPA-induced insomnia, offering promising insights into potential therapeutic applications for hedonistic behavior.

### DSIP-CBBBP and DSIP improve sleep and pleasure associated biochemical biomarkers

**FIGURE 6:**
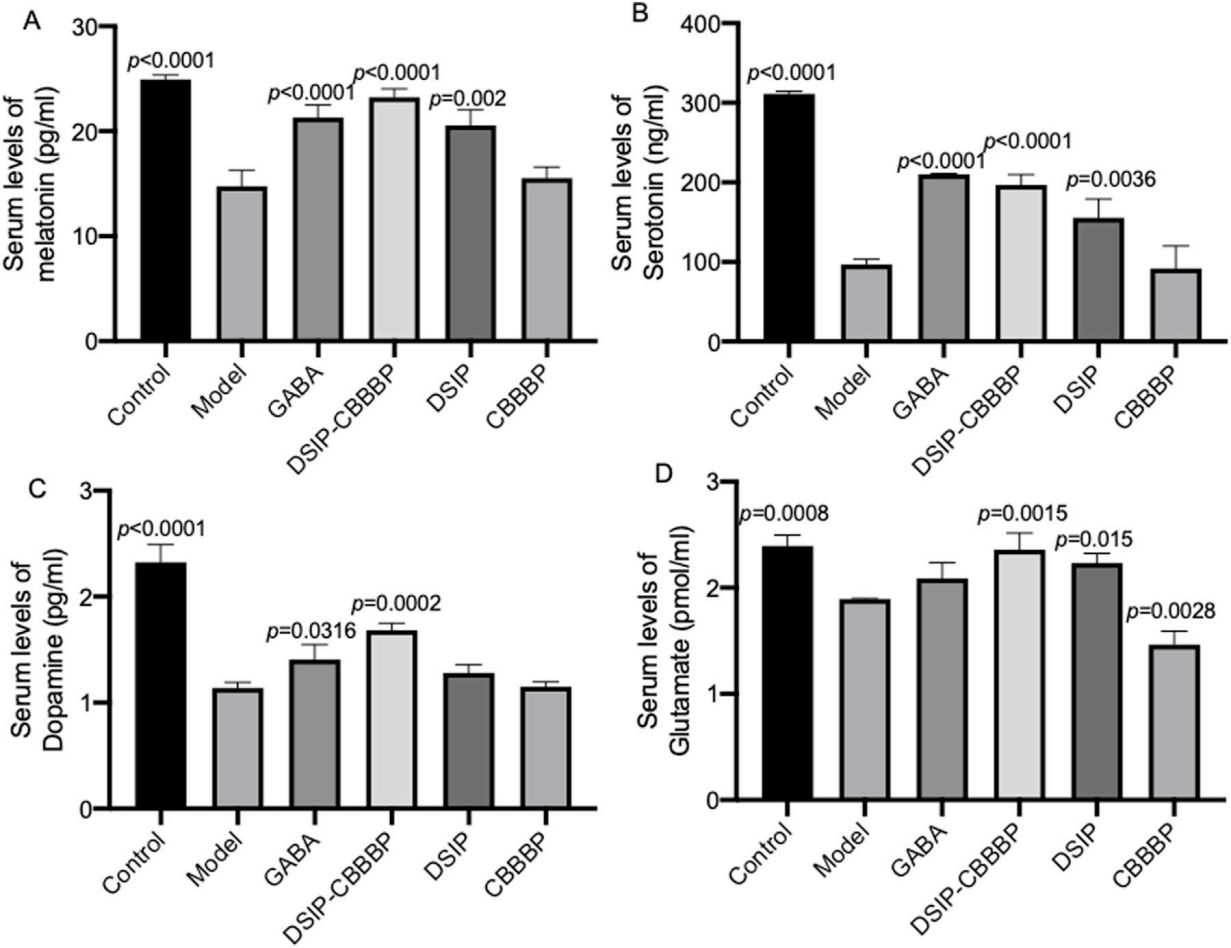
Different biochemical markers measured across various groups of mice. **(A)**, serum levels of melatonin. **(B)**, serum levels of serotonin. **(C)**, serum levels of dopamine. **(D)**, serum levels of glutamate. Control (healthy), Model (insomnia induced by PCPA), DSIP (treated with DSIP peptide), DSIP-CBBBP (treated with fusing peptides DSIP and CBBBP), and CBBBP. N = 6 for each group. The statistical difference is significant if *p* < 0.05 via the model group.

Figure 6A shows the levels of serum melatonin among different groups (F (5, 12) = 38.31, *p* < 0.0001), reducing in the model when compared with the control group which presents baseline melatonin levels (*p* < 0.0001). DSIP and DSIP-CBBBP treatments both result in increased melatonin levels (*p* < 0.05 for both) compared to the model group, indicating a potential stress-mitigating effect. However, CBBBP treatment does not significantly reduce melatonin levels compared to the model group (*p* = 0.869), suggesting less efficacy in countering stress-induced changes. Figure 6B depicts serum serotonin among different groups (F (5, 12) = 76.27, *p* < 0.0001), and exhibits significantly lower levels in the model when compared with the control group which has baseline levels (*p* < 0.0001), indicative of sleep disorder. Both DSIP and DSIP-CBBBP treatments show increased serotonin levels (*p* < 0.0001), pointing to their potential improve sleep effects. The CBBBP group also does not show effects on the serotonin levels (*p* = 0.994), suggesting that CBBBP peptide alone cannot affect sleep quality. Figure 6C illustrates serum dopamine levels among different groups (F (5, 12) = 57.41, *p* < 0.0001), the neurotransmitter associated with pleasure, wakefulness and alertness. The Model group has significantly lower dopamine levels (*p* < 0.0001), which could be associated with neuronal stress or damage. Both GABA and DSIP-CBBBP treatments result in higher dopamine levels (*p* < 0.05) compared to the model group, suggesting a neuroprotective or restorative effect. The CBBBP and DSIP group, however, shows no significant difference (*p* > 0.05) from the model group, indicating that CBBBP or DSIP peptide alone may not be sufficient to alter dopamine levels in this context. Figure 6D presents serum glutamate levels among different groups (F (5, 12) = 27.42, *p* < 0.0001), which are indicative of cognition, memory, and learning. The Control group has normal glutamate levels. The model group shows reduced levels of glutamate (*p* = 0.0008), suggesting that the model impacts brain function. GABA treatment does not significantly alter glutamate levels (*p* = 0.2003). CBBBP group shows significantly reduced glutamate levels (*p* = 0.0028) compared to the model, which might raise concerns about brain function with this treatment.

### DSIP-CBBBP showed the most substantial reduction in wakefulness time

**FIGURE 7:**
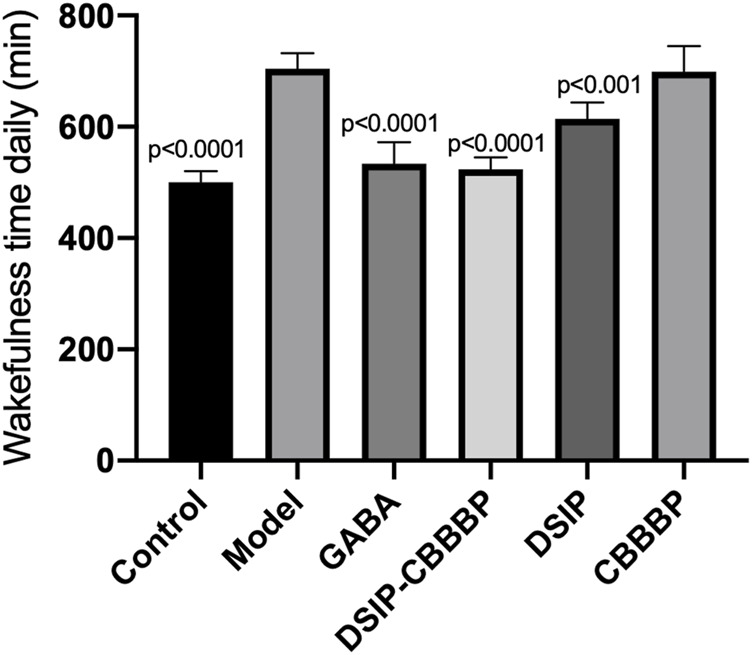
Effect of different agents on wakefulness time in insomnia mice. This figure illustrates the wakefulness time (in minutes per day) of mice across six groups: Control, Model, GABA, DSIP-CBBBP, DSIP, and CBBBP. After a 5-day PCPA-induced insomnia model was established, treatments were administered over a 7-day period. N = 6 for each group. The statistical difference is significant if *p* < 0.05 via model group.

There were significant differences for wakeful time daily among different group (F (5, 30) = 47.59, *p* < 0.0001, Figure 7). The control group exhibited an average wakefulness time of 480 ± 30 min per day, reflecting normal sleep-wake cycles. The model group, induced with PCPA to simulate insomnia, showed a significant increase in wakefulness time to 720 ± 45 min per day, indicating severe insomnia. Mice treated with GABA had an average wakefulness time of 510 ± 45 min per day, significantly reducing wakefulness compared to the model group (*p* < 0.0001). The DSIP-CBBBP treated group exhibited an average wakefulness time of 500 ± 32 min per day, showing a marked improvement in reducing insomnia symptoms compared to the model group (*p* < 0.0001). The DSIP treated group showed an average wakefulness time of 600 ± 40 min per day, which was significantly different from the model group (*p* < 0.001). The CBBBP treated group had an average wakefulness time of 690 ± 42 min per day, showing no significant difference from the model group (*p* > 0.05). These results indicate that treatment with GABA, DSIP-CBBBP and more effectively reduced wakefulness time, with DSIP-CBBBP showing the most substantial reduction, suggesting its potential as a therapeutic agent for mitigating insomnia.

### DSIP-CBBBP improves the hippocampus tissues in insomnia models

**FIGURE 8:**
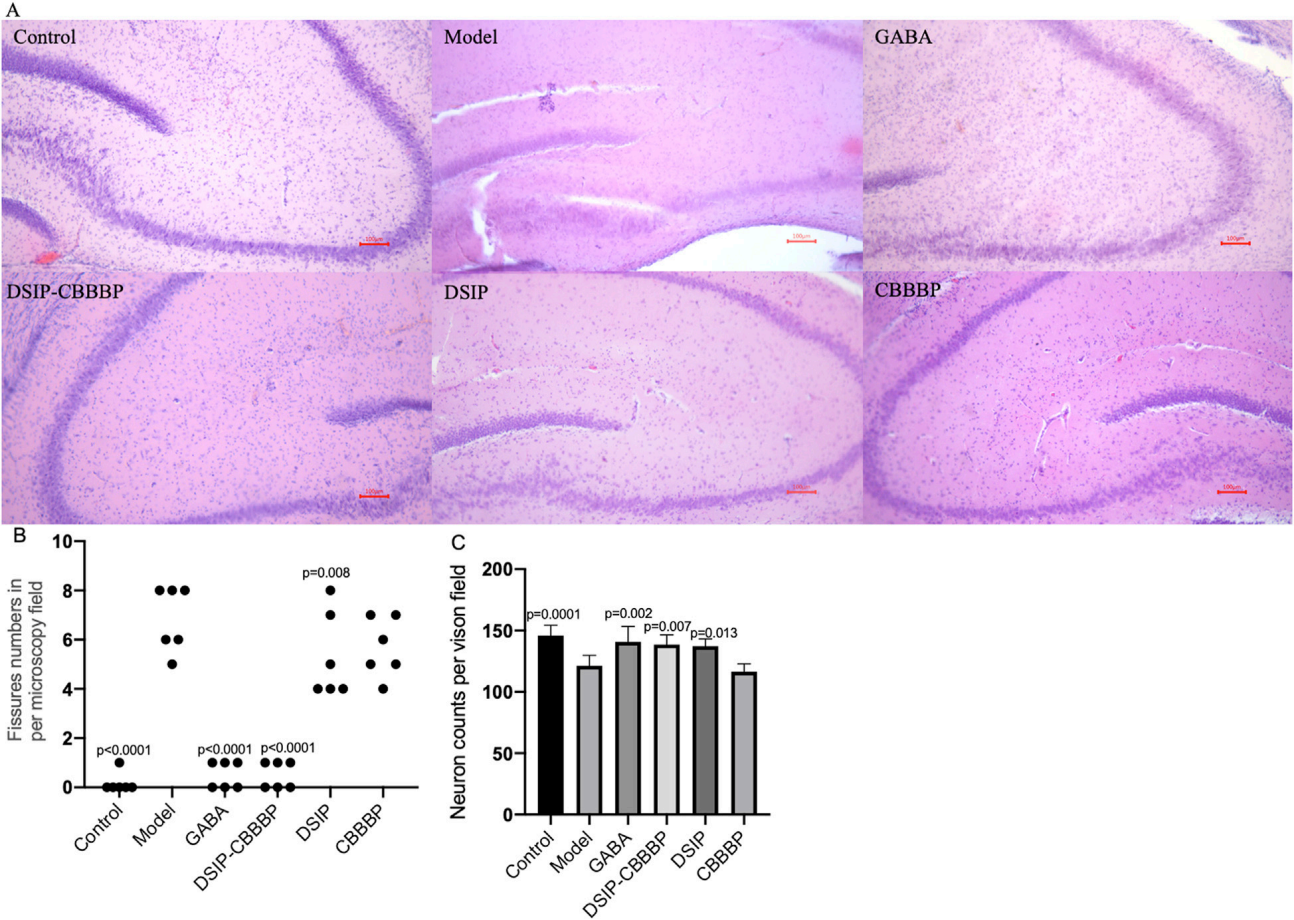
Brain tissue analysis. **(A)**, Histological sections of a mouse brain stained with hematoxylin and eosin (H&E). **(B)**, the fissure numbers in different groups. **(C)**, the neuron density in different groups. Control, healthy mice. Model, insomnia mice induced by PCPA. The model mice treated with GABA, DSIP, CBBBP and DSIP-CBBBP fusion peptides. N = 6 for each group. The statistical difference is significant if *p* < 0.05 via the model group.

H&E analysis shows the hippocampus tissues in perfect condition in healthy controls. In the model with insomnia induced by PCPA shows the fissure in the tissues. DSIP-CBBBP have inhibitory functions on the symptoms. On the other hand, DSIP and CBBBP show no improvement when compared with the model group (Figure 8A). The analysis of fissure numbers per microscopy field across different experimental groups reveals significant variations in brain tissue morphology (Figure 8B, F (5, 30) = 48.51, *p* < 0.0001). The PCPA-induced insomnia model group exhibited a high number of fissures. Treatment with GABA showed a statistically significant reduction (*p* < 0.0001) when compared with the model group. Similarly, DSIP treatment and CBBBP treatment resulted cannot change the number significantly (*p* > 0.05). Notably, the DSIP-CBBBP fusion peptide group exhibited the most substantial reduction (*p* < 0.0001), demonstrating a highly significant difference compared to the model group. These findings highlight the therapeutic potential of DSIP-CBBBP fusion peptide with the most pronounced effect.

Figure 8C presents the impact of different treatments on neuro density across experimental groups (F(5, 30) = 11.15, *p* < 0.0001). The control group exhibits the highest neuron density, representing the baseline levels expected in healthy conditions. In contrast, the model group shows a significant reduction in neuron density (*p* = 0.0001), highlighting the detrimental effects of sleep deprivation on brain health. Treatment with GABA significantly restores neuron density compared to the model group, as evidenced by the large improvement and the (*p* = 0.002), underscoring its neuroprotective effects. The DSIP-CBBBP and DSIP groups demonstrates the most substantial increase in neuron density, indicating a highly significant difference compared to the model group (*p* = 0.007 and 0.013, respectively). These results highlight the therapeutic potential of DSIP-CBBBP in ameliorating neuronal loss associated with insomnia, suggesting promising avenues for developing treatments for sleep-related neurological impairments.

## Discussion

The study demonstrated the significant potential of DSIP-CBBBP fusion peptides in promoting sleep and balancing neurotransmitters, using a PCPA-induced insomnia mouse model. Results indicate that DSIP-CBBBP effectively modulates neurotransmitter levels, particularly 5-HT, glutamate, dopamine, and melatonin, leading to improved sleep quality compared to DSIP alone. This peptide’s ability to restore neurotransmitter balance highlights its promise as a novel therapeutic agent for sleep disorders. Additionally, the findings reveal that DSIP-CBBBP crosses the blood-brain barrier more effectively than traditional peptides, potentially enhancing its efficacy *in vivo*. The study supports the utility of DSIP-CBBBP in developing therapies targeting neurotransmitter dysregulation and sleep disturbances, offering a promising avenue for future research in sleep-related treatments.

The differential outcomes observed in Figure 3 suggest that the anxiolytic efficacy of the treatment’s hinges on their specific molecular configurations and the mechanisms by which they modulate neurobiological pathways. Specifically, the enhanced anxiolytic effects seen with DSIP-CBBBP treatment compared to DSIP alone likely arise from the synergistic role of CBBBP and DSIP. This synergy potentially improves the bioavailability or targeting of the fusion protein, thereby amplifying its therapeutic effects. The CBBBP component could enhance the penetration of DSIP-CBBBP across BBB or improve its interactions with neural substrates involved in anxiety modulation. This is particularly relevant as the BBB represents a major challenge in CNS drug delivery, often preventing neurotherapeutic agents from effectively reaching the brain. The utilization of brain-penetrating molecular transport vectors like CBBBP, which can transport drugs into the brain via receptor-mediated transcytosis—a natural mechanism for BBB crossing—underscores a significant advancement in overcoming this barrier. This approach not only facilitates the delivery of DSIP into the brain but also highlights the potential of brain-homing peptides as effective carriers for small molecule drugs, targeting brain parenchyma directly and efficiently. It sheds light on the emerging knowledge regarding the peptides, their specific receptors on brain endothelial cells, the selection of drug payloads, the design of DSIP-CBBBP, mechanisms of brain entry, and the efficiency of delivery to the brain (Lv et al., 2021).

On the other hand, the lack of a significant anxiolytic effect with GABA treatment raises questions about its bioavailability or receptor interactions in the specific context of the EPM test, possibly due to differences in the route of administration, dosage, or the animal model’s specific pathology. The absence of a significant effect with CBBBP alone suggests that the CBBBP construct may require the DSIP component to engage with the relevant neural pathways effectively. Overall, these findings underscore the complexity of the molecular interactions with the neural circuitry underlying anxiety and highlight the necessity for a multifaceted approach in the design of anxiolytic treatments.

The results from the tail suspension test, as depicted in Figure 4, provide compelling evidence on the effects of different treatments on depressive-like behaviors in an animal model. This test, which evaluates depressive states through the analysis of immobility and struggle times, demonstrated that the control group displayed minimal depressive-like behaviors, as evidenced by the lowest percentage of immobility. Conversely, the model group exhibited a significant elevation in immobility time, indicative of an insomnia-induced depressive-like state. Notably, treatment with GABA and specific interventions using DSIP-CBBBP and DSIP markedly decreased immobility times compared to the model group, suggesting their effectiveness in mitigating depressive-like behaviors. DSIP’s effect, while significant, was less pronounced than that of DSIP-CBBBP. Furthermore, Figure 4B’s analysis of struggle time percentages reinforce these findings; the control group showed the highest struggle times, correlating with lesser depressive-like behaviors. In contrast, the model group’s reduced struggle time aligned with increased depressive-like conditions. However, treatments with GABA, DSIP-CBBBP, and DSIP all resulted in increased struggle times compared to the model group, with CBBBP alone not showing a similar effect, indicating a differential impact on depressive-like behaviors and highlighting the potential therapeutic value of these treatments in addressing depressive symptoms associated with insomnia (Riemann et al., 2020; Vargas and Perlis, 2020).

Figure 5 highlights the substantial influence of peptide treatments on the sucrose preference in mice disrupted by PCPA. The significant variation among the groups underscores the differential impacts of the treatments. Notably, the control group retained baseline sucrose preference levels, whereas the model group exhibited a significant reduction, underscoring the effect of PCPA. Treatments with DSIP and the DSIP-CBBBP fusion peptide significantly countered this reduction, showcasing their potential as therapeutic agents for modulating hedonistic behavior. The DSIP-CBBBP group, in particular, showed superior improvement over both the model and DSIP-only groups, suggesting enhanced efficacy of the fusion peptide. Conversely, the CBBBP peptide alone demonstrated a less marked effect, indicating that the primary therapeutic benefits are attributable to the DSIP component. These findings suggest that DSIP, especially when combined with CBBBP, effectively mitigates PCPA-induced changes in sucrose preference, offering valuable insights into potential treatments for hedonistic behavior modifications in clinical settings (Markov, 2022; Pak et al., 2022).

The research presented in Figures 6A–D explores the impact of various treatments on biochemical biomarkers associated with stress and sleep disorders, highlighting the differential efficacy of these interventions in modulating serum levels of melatonin, serotonin, dopamine, and glutamate among different groups. Specifically, the study reveals a significant reduction in serum melatonin levels in the model group compared to the control, suggesting the induction of stress. Treatments with DSIP and DSIP-CBBBP are shown to effectively increase melatonin levels, indicating a stress-mitigating potential, whereas CBBBP treatment alone does not significantly affect melatonin levels, pointing to its limited efficacy in this context. Similarly, serotonin levels are significantly lower in the model group, indicating a potential sleep disorder. Both DSIP and DSIP-CBBBP treatments increase serotonin levels significantly, suggesting their effectiveness in improving sleep effects, while CBBBP alone shows no significant impact on serum levels of melatonin, serotonin, dopamine, and glutamate.

Melatonin plays a crucial role in regulating the sleep-wake cycle, with its production naturally declining with age, correlating with a decrease in sleep quality among older adults, suggesting a potential therapeutic role in treating sleep disorders through supplementation. Despite its theoretical appeal for treating insomnia (Choi et al., 2022), especially as an age-related intervention (Xu et al., 2020), the efficacy, optimal dosing, and pharmaceutical formulations of melatonin remain under-researched, indicating a need for further scientific validation to establish its clinical utility (Poza et al., 2022). Serotonin, a neurotransmitter primarily known for its role in mood regulation, also appears to have a significant impact on sleep and breathing parameters, as observed in this study. The observed correlations suggest that lower levels of serum serotonin are associated with more severe sleep-disordered breathing symptoms, including apnea and oxygen desaturation, indicating its potential role in the pathophysiology of sleep-related breathing disorders (Wieckiewicz et al., 2023). Serotonin, a key neurotransmitter involved in mood regulation and sleep, plays a crucial role in the complex interplay between insomnia and depression. Its regulation affects sleep continuity, REM sleep patterns, and has implications for the therapeutic mechanisms of antidepressants, highlighting its importance in both the manifestation of insomnia and its potential as a target for preventing depression linked to sleep disturbances (Riemann et al., 2020).

Dopamine plays a crucial role in the transition from non–rapid eye movement (NREM) sleep to rapid eye movement (REM) sleep through a transient increase in the basolateral amygdala (BLA), acting on D2 dopamine receptors to initiate the NREM-to-REM shift. This DA signaling mechanism in the BLA not only facilitates the normal sleep cycle but also contributes to the occurrence of cataplectic attacks in narcoleptics, illustrating its significance in both physiological and pathological aspects of sleep regulation (Hasegawa et al., 2022). Dopamine plays a crucial role in modulating the reward network in chronic insomnia disorder (CID), particularly in the context of comorbid depression, where genetic variations related to DA can differentially affect functional connectivity within the nucleus accumbens and broader reward networks. These variations influence the balance between different network activities, leading to altered responses in the salience, visual, internal reward, and executive control networks, thereby highlighting the complex interplay between DA, reward network dysregulation, and the manifestation of depressive symptoms in CID patients (Gong et al., 2023). Glutamate, a key neurotransmitter involved in the pathophysiology of schizophrenia and the regulation of sleep-wake neurobiology, plays a crucial role in mediating the quality of sleep, as evidenced by the correlation between sleep disturbances and glutamate levels in specific brain regions of individuals with schizophrenia. This relationship suggests that interventions targeting glutamatergic system modulation could enhance sleep quality and, potentially, alleviate the severity of positive symptoms in schizophrenia, highlighting the interconnectedness of sleep regulation and glutamate’s function in mental health disorders (Korenic et al., 2020). The study utilizing single-voxel proton magnetic resonance spectroscopy (1H MRS) to analyze glutamate levels relative to creatine in individuals with Primary Insomnia (PI) suggests no significant direct association between glutamate concentrations in the dorsal anterior cingulate cortex (dACC) and measures of sleep quality within subjects with Major Depressive Disorder (MDD). However, the lack of a significant contribution of insomnia severity to the observed glutamatergic measures in MDD indicates that while glutamate plays a critical role in the pathophysiology of MDD, its specific relationship with insomnia (Benson et al., 2020).

Our analysis of dopamine and glutamate levels provides insights into the neuroprotective and cognitive effects of the treatments. Dopamine, a neurotransmitter associated with pleasure, wakefulness, and alertness, is found at significantly lower levels in the model group, which could be indicative of neuronal stress or damage. The application of GABA and DSIP-CBBBP treatments increases dopamine levels, suggesting a potential neuroprotective or restorative effect, whereas CBBBP and DSIP treatments do not show a significant impact on dopamine levels, indicating their insufficiency in altering dopamine concentrations in this context. Glutamate levels, crucial for cognition, memory, and learning, are reduced in the model group, implying an adverse effect on brain function. Interestingly, while GABA treatment does not significantly change glutamate levels, CBBBP treatment reduces them further, raising concerns regarding the potential negative impact on brain function with this specific treatment. Collectively, these findings underscore the complexity of treating stress and sleep disorders, highlighting the variable effectiveness of treatments like GABA, DSIP-CBBBP, DSIP, and CBBBP alone in modulating key biochemical biomarkers.

The analysis of fissure numbers in brain tissue across different experimental groups offers valuable insights into the structural effects of various treatments on insomnia-induced changes. The significant increase in fissures observed in the PCPA-induced insomnia model group suggests that sleep disturbances may lead to notable changes in brain tissue morphology (Figures 8A,B). Conversely, the significant reduction in fissures with GABA treatment suggests that GABA may help mitigate these actual tissue stress. The most notable reduction in fissures with the DSIP-CBBBP fusion peptide group highlights its potential efficacy in improving brain tissue morphology. The analysis of neuron density further supports these findings, with the model group showing a marked reduction in neuron density (Figure 8C), reflecting the adverse effects of sleep deprivation on neuronal health. The restoration of neuron density with GABA treatment underscores its neuroprotective properties, suggesting a protective effect against neuronal loss. DSIP-CBBBP fusion peptide and DSIP treatments demonstrated even more significant increases in neuron density compared to the model group, emphasizing the potential of these treatments in counteracting neuronal loss associated with insomnia. These results underscore the promising therapeutic potential of DSIP-CBBBP in mitigating sleep-related neurological impairments, paving the way for future research into targeted treatments for such conditions.

## Data Availability

The raw data supporting the conclusions of this article will be made available by the authors, without undue reservation.

## References

[B1] Abdou A. M. Higashiguchi S. Horie K. Kim M. Hatta H. Yokogoshi H. (2006). Relaxation and immunity enhancement effects of γ‐aminobutyric acid (GABA) administration in humans. Biofactors 26 (3), 201–208. 10.1002/biof.5520260305 16971751

[B2] Al-Kuraishy H. M. Al-Gareeb A. I. Saad H. M. Batiha G. E.-S. (2023). Benzodiazepines in Alzheimer’s disease: beneficial or detrimental effects. Inflammopharmacology 31 (1), 221–230. 10.1007/s10787-022-01099-4 36418599

[B3] Becker M. L. Remsen E. E. Pan D. Wooley K. L. (2004). Peptide-derivatized shell-cross-linked nanoparticles. 1. Synthesis and characterization. Bioconjugate Chem. 15 (4), 699–709. 10.1021/bc049946e 15264856

[B4] Benson K. L. Bottary R. Schoerning L. Baer L. Gonenc A. Eric Jensen J. (2020). 1H MRS measurement of cortical GABA and glutamate in primary insomnia and major depressive disorder: relationship to sleep quality and depression severity. J. Affect. Disord. 274, 624–631. 10.1016/j.jad.2020.05.026 32663996 PMC10662933

[B5] Chen Y. Li S. Zhao J. Cao X. Wang F. (2022). Efficient drug delivery by novel cell-penetrating peptide derived from Midkine, with two heparin binding sites braced by a length-specific helix. J. Drug Target. 30 (3), 326–333. 10.1080/1061186X.2021.1999960 34708678

[B6] Choi K. Lee Y. J. Park S. Je N. K. Suh H. S. (2022). Efficacy of melatonin for chronic insomnia: systematic reviews and meta-analyses. Sleep. Med. Rev. 66, 101692. 10.1016/j.smrv.2022.101692 36179487

[B7] Choudhury M. E. Mikami K. Nakanishi Y. Matsuura T. Utsunomiya R. Yano H. (2022). Insomnia and depressive behavior of MyD88-deficient mice: relationships with altered microglial functions. J. Neuroimmunol. 363, 577794. 10.1016/j.jneuroim.2021.577794 34971898

[B8] Frøjd L. A. Munkhaugen J. Moum T. Sverre E. Nordhus I. H. Papageorgiou C. (2021). Insomnia in patients with coronary heart disease: prevalence and correlates. J. Clin. Sleep Med. 17 (5), 931–938. 10.5664/jcsm.9082 33399066 PMC8320477

[B9] Girardeau G. Lopes-Dos-Santos V. (2021). Brain neural patterns and the memory function of sleep. Science. 374 (6567), 560–564. 10.1126/science.abi8370 34709916 PMC7611961

[B10] Gong L. Chen K. Zhang H. Zhang S. Xu R. Liu D. (2023). Dopamine multilocus genetic profile influence on reward network in chronic insomnia disorder with depression. Sleep. Med. 112, 122–128. 10.1016/j.sleep.2023.09.026 37839273

[B11] Hasegawa E. Miyasaka A. Sakurai K. Cherasse Y. Li Y. Sakurai T. (2022). Rapid eye movement sleep is initiated by basolateral amygdala dopamine signaling in mice. Science 375 (6584), 994–1000. 10.1126/science.abl6618 35239361

[B12] Hashemi Y. H. Heiat M. Alavian S. M. Rezaei E. (2022). A new combination: anti glypican-3 scFv and diphtheria toxin with the best flexible linker. Protein J. 41 (4-5), 527–542. 10.1007/s10930-022-10074-5 36001255

[B13] Hepsomali P. Groeger J. A. Nishihira J. Scholey A. (2020). Effects of oral gamma-aminobutyric acid (GABA) administration on stress and sleep in humans: a systematic review. Front. Neurosci. 14, 923. 10.3389/fnins.2020.00923 33041752 PMC7527439

[B14] Hong J. Chen J. Kan J. Liu M. Yang D. (2020). Effects of acupuncture treatment in reducing sleep disorder and gut microbiota alterations in PCPA-induced insomnia mice. Evidence-Based Complementary Altern. Med. 2020, 14. 10.1155/2020/3626120 PMC7647758 33178314

[B15] Huang B. Liang S. Li X. Xie Z. Yang R. Sun B. (2024). Postweaning intermittent sleep deprivation enhances defensive attack in adult female mice via the microbiota-gut-brain axis. Prog. Neuro-Psychopharmacology Biol. Psychiatry 130, 110915. 10.1016/j.pnpbp.2023.110915 38104921

[B16] Kapsi S. Katsantoni S. Drigas A. (2020). The role of sleep and impact on brain and learning. Int. J. Recent Contrib. Eng. Sci. IT 8 (3), 59–68. 10.3991/ijes.v8i3.17099

[B17] Knowlden A. Grandner M. (2021). 138 short sleep and insomnia as independent predictors of obesity, hypertension, and diabetes. Sleep 44, A56. 10.1093/sleep/zsab072.137

[B18] Korenic S. A. Klingaman E. A. Wickwire E. M. Gaston F. E. Chen H. Wijtenburg S. A. (2020). Sleep quality is related to brain glutamate and symptom severity in schizophrenia. J. psychiatric Res. 120, 14–20. 10.1016/j.jpsychires.2019.10.006 31610406

[B19] Lavigna G. Masone A. Bouybayoune I. Bertani I. Lucchetti J. Gobbi M. (2021). Doxycycline rescues recognition memory and circadian motor rhythmicity but does not prevent terminal disease in fatal familial insomnia mice. Neurobiol. Dis. 158, 105455. 10.1016/j.nbd.2021.105455 34358614 PMC8463834

[B21] Lv Y.-b. Zhou Q. Yan J.-x. Luo L.-s. Zhang J.-l. (2021). Enzymolysis peptides from Mauremys mutica plastron improve the disorder of neurotransmitter system and facilitate sleep-promoting in the PCPA-induced insomnia mice. J. Ethnopharmacol. 274, 114047. 10.1016/j.jep.2021.114047 33753142

[B22] Maiolino G. Bisogni V. Soranna D. Pengo M. F. Pucci G. Vettor R. (2021). Effects of insomnia and restless legs syndrome on sleep arterial blood pressure: a systematic review and meta-analysis. Sleep. Med. Rev. 59, 101497. 10.1016/j.smrv.2021.101497 34044356

[B23] Markov D. D. (2022). Sucrose preference test as a measure of anhedonic behavior in a chronic unpredictable mild stress model of depression: outstanding issues. Brain Sci. 12 (10), 1287. 10.3390/brainsci12101287 36291221 PMC9599556

[B24] Morgan H. (2023). Depression research and DSIP peptide. Health.

[B25] Nader D. Gowing L. (2020). Is long-term benzodiazepine use a risk factor for cognitive decline? Results of a systematic review. J. Addict. 2020, 1569456. 10.1155/2020/1569456 32047702 PMC7001667

[B26] Pak K. Seo S. Lee M. J. Kim K. Suh S. Lee J. (2022). Hedonic rating of sucrose is sub-regionally associated with striatal dopamine transporter in humans. Neuroendocrinology 112 (4), 338–344. 10.1159/000517319 34034262

[B27] Poza J. J. Pujol M. Ortega-Albás J. J. Romero O. Insomnia Study Group of the Spanish Sleep Society SES (2022). Melatonin in sleep disorders. Neurol. Engl. Ed. 37 (7), 575–585. 10.1016/j.nrleng.2018.08.004 36064286

[B28] Riemann D. Krone L. B. Wulff K. Nissen C. (2020). Sleep, insomnia, and depression. Neuropsychopharmacology 45 (1), 74–89. 10.1038/s41386-019-0411-y 31071719 PMC6879516

[B29] Rourke E. Law F. (2021). Addiction to prescription medication: benzodiazepines, z‐drugs and gabapentinoids. Cambridge University Press, 68–96.

[B30] Tyagi P. Banerjee R. Basu S. Yoshimura N. Chancellor M. Huang L. (2006). Intravesical antisense therapy for cystitis using TAT-peptide nucleic acid conjugates. Mol. Pharm. 3 (4), 398–406. 10.1021/mp050093x 16889433

[B31] Vargas I. Perlis M. L. (2020). Insomnia and depression: clinical associations and possible mechanistic links. Curr. Opin. Psychol. 34, 95–99. 10.1016/j.copsyc.2019.11.004 31846870 PMC12172691

[B20] Wang L. Wang X. Wang P. Li C. Chen Y. Chen Y. (2022). Regulatory effects of Pinellia-Coix Seed on hippocampal orexin, its receptors and serum cytokines in PCPA insomnia model rats. Acad. J. Chin. PLA Med. Sch. 43 (4), 472–478. 10.3969/j.issn.2095-5227.2022.04.019

[B32] Wieckiewicz M. Martynowicz H. Lavigne G. Lobbezoo F. Kato T. Winocur E. (2023). An exploratory study on the association between serotonin and sleep breathing disorders. Sci. Rep. 13 (1), 11800. 10.1038/s41598-023-38842-y 37479853 PMC10362063

[B33] Xu H. Zhang C. Qian Y. Zou J. Li X. Liu Y. (2020). Efficacy of melatonin for sleep disturbance in middle-aged primary insomnia: a double-blind, randomised clinical trial. Sleep. Med. 76, 113–119. 10.1016/j.sleep.2020.10.018 33157425

[B34] Yamatsu A. Yamashita Y. Maru I. Yang J. TatsuzakI J. Kim M. (2015). The improvement of sleep by oral intake of GABA and apocynum venetum leaf extract. J. Nutr. Sci. vitaminology 61 (2), 182–187. 10.3177/jnsv.61.182 26052150

[B35] Yoto A. Murao S. Motoki M. Yokoyama Y. Horie N. Takeshima K. (2012). Oral intake of γ-aminobutyric acid affects mood and activities of central nervous system during stressed condition induced by mental tasks. Amino Acids 43 (3), 1331–1337. 10.1007/s00726-011-1206-6 22203366

